# Functional neurological disorder: an evolutionary perspective

**DOI:** 10.1093/emph/eoaf036

**Published:** 2025-12-12

**Authors:** Akihiro Nishi, Jon Stone

**Affiliations:** Department of Epidemiology, Fielding School of Public Health, University of California, Los Angeles, 650 Charles E Young Dr. S, Los Angeles, CA 90095, USA; California Center for Population Research, University of California, Los Angeles, 337 Charles E Young Dr E, Los Angeles, CA 90095, USA; Centre for Clinical Brain Sciences, University of Edinburgh, Royal Infirmary of Edinburgh, Edinburgh EH16 4SA, UK

Functional neurological disorder (FND) is among the commonest disorders in neurological practice with an estimated prevalence of 80–140 per 100 000 individuals and a female predominance of ~70% [1]. It is characterized by symptoms involving an excess or decrease in voluntary movement or sensory pathways such as seizures, paralysis, tremor, and visual loss leading to disability and distress [1]. FND has been documented worldwide since antiquity [1, 2]. Formerly known as hysteria and conversion disorder, FND lies at the neurology-psychiatry interface and has long been stigmatized and neglected. In recent years, the field has undergone a renaissance, with a growing consensus that FND has characteristic clinical features that allow a rule-in diagnosis [2].

Magnetic resonance imaging (MRI) and other investigations are typically normal, but functional MRI and neurophysiological studies shed light on new underlying mechanisms of FND [1, 3]. At the brain network level, data suggest a breakdown in communication between the recently evolved prefrontal cortex and more conserved brain regions like amygdala, periaqueductal gray (PAG), and insula, governing threat detection. FND symptoms are hypothesized to arise from mismatches between predicted and ‘bottom-up’ sensory experience, with associated disruption in sense of agency (the feeling that one has initiated and is in control of one’s own movements and their outcomes) [1, 3]. This pattern is distinct from feigning [2]. Psychosocial mechanisms—sometimes related to stress and past adversity—also play an important role in many people with FND.

Some studies have explored genes potentially involved in FND (e.g. *TPH2* in the serotonin pathway) [4]. In addition, many common FND comorbidities such as anxiety and nociplastic pain (pain from chronic alteration of central nervous system processing, not from inflammation or injury [5]) have a strong genetic basis. However, genome-wide association studies specific to FND are lacking, and there is no evidence that any gene involved in FND has undergone a selective sweep (a sign of past natural selection).

## Figures and Tables

**Figure 1:**
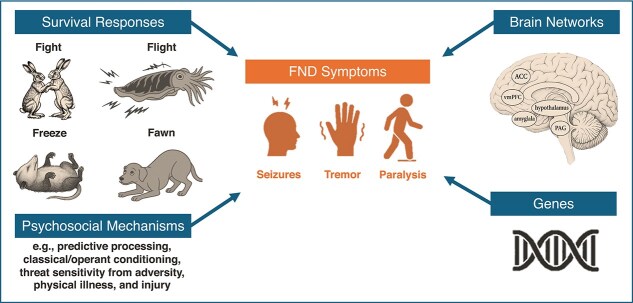
Evolutionary understanding of FND symptoms. ACC: Anterior cingulate cortex, vmPFC: Ventromedial prefrontal cortex, and PAG: Periaqueductal gray.

## EVOLUTIONARY PERSPECTIVES

Researchers have hypothesized that FND symptoms originate from several *evolved defensive strategies* [6] (Fig. 1). A primary evolved defense mechanism is *freeze*, part of the classic fight-flight-freeze-fawn spectrum triggered by predatory imminence [6]. Freeze involves coordinated activity across limbic and brainstem circuits, including the amygdala and PAG, and may be associated with motor inhibition and autonomic changes (often characterized by parasympathetic dominance), although profiles can be mixed [7]. This tonic immobility is widely observed in rats, squid, and other animals. In FND, this freeze-like reaction, which maps on to limb paralysis or some ‘motionless unresponsive’ events—is often precipitated by exposure to various physical or psychological threats—such as pain, injury, infections, faints, panic or psychological stress. These responses then become ‘fixed’, implying a system failure to return to a homeostatic baseline after the threat has passed, perhaps through maladaptive predictive processing [1, 2]. By contrast, hyperkinetic FND symptoms such as tremor and seizures map better onto a mixed *freeze* and *fight (or fawn) response* rather than *freeze* alone.

Animal models for FND have not been explored systematically but there are potentially similar behaviors in chimpanzees, elephants and mice, as well as learned forms of paralysis, for example in dogs learning to beg [8].

## FUTURE IMPLICATIONS

Viewing FND through an evolutionary lens could illuminate new approaches to understanding its mechanisms and treatment. Recognizing that FND symptoms may arise, in part, from deeply rooted survival responses, involving coordinated motor and autonomic changes, can have implications for how clinicians communicate with patients and their families, and how therapies are developed. Finally, large-scale studies of gene–brain network interactions (using biobank data [5]), along with detailed research into the pathophysiology of FND episodes, offer a promising path forward.
